# Performance of SOBA-AD blood test in discriminating Alzheimer’s disease patients from cognitively unimpaired controls in two independent cohorts

**DOI:** 10.1038/s41598-024-57107-w

**Published:** 2024-04-04

**Authors:** Amy Chen, Dylan Shea, Valerie Daggett

**Affiliations:** 1 AltPep Corporation, 1150 Eastlake Avenue N, Suite 800, Seattle, WA 98109 USA; 2 University of Washington, Box 355610, Seattle, WA 98195-5610 USA

**Keywords:** Neurodegenerative diseases, Alzheimer's disease, Diagnostic markers, Biomarkers, Peptides

## Abstract

Amyloid-beta (Aβ) toxic oligomers are critical early players in the molecular pathology of Alzheimer’s disease (AD). We have developed a Soluble Oligomer Binding Assay (SOBA-AD) for detection of these Aβ oligomers that contain α-sheet secondary structure that discriminates plasma samples from patients on the AD continuum from non-AD controls. We tested 265 plasma samples from two independent cohorts to investigate the performance of SOBA-AD. Testing was performed at two different sites, with different personnel, reagents, and instrumentation. Across two cohorts, SOBA-AD discriminated AD patients from cognitively unimpaired (CU) subjects with 100% sensitivity, > 95% specificity, and > 98% area under the curve (AUC) (95% CI 0.95–1.00). A SOBA-AD positive readout, reflecting α-sheet toxic oligomer burden, was found in AD patients, and not in controls, providing separation of the two populations, aside from 5 SOBA-AD positive controls. Based on an earlier SOBA-AD study, the Aβ oligomers detected in these CU subjects may represent preclinical cases of AD. The results presented here support the value of SOBA-AD as a promising blood-based tool for the detection and confirmation of AD.

## Introduction

An accurate and earlier molecular diagnosis of Alzheimer’s Disease (AD) should enable earlier care and the chance for disease-modifying therapies to intervene before irreversible neuronal damage occurs. Substantial evidence implicates the formation of soluble oligomers of the amyloid-beta peptide (Aβ, 42 amino acid fragment) as the earliest event in the molecular pathophysiology of AD^1–8^. These soluble Aβ oligomers trigger a cascade of events, including disruption of synaptic transmission, aggregation to form fibrils and plaques, tau hyperphosphorylation, tau neurofibrillary tangle formation, neuroinflammation, and neurodegeneration^5–12^.

Through atomistic molecular dynamics simulations of unrelated amyloidogenic proteins, we discovered a defining feature of toxic oligomers: a nonstandard protein secondary structure called alpha-sheet (α-sheet)^13–22^. Outwardly, α-sheets resemble β-sheets except that the carbonyl oxygens are aligned on one face of a strand and the NH groups on the other instead of alternating, giving rise to different physical properties^15,17^. Analysis of toxic in vitro prepared Aβ oligomers by a variety of spectroscopic methods confirmed the presence of α-sheet structure in hexameric and dodecameric oligomer aggregates^20^. This finding was confirmed in cerebrospinal fluid (CSF) from AD patients and the lack of such oligomers in cognitively unimpaired (CU) controls^22^. The nonstandard structure of the α-sheet oligomers represents a unique target for detection both earlier and throughout the AD continuum.

There remain limitations to differential diagnosis of AD by cognitive function exams, amyloid PET imaging, and more recently, CSF and blood biomarkers. In primary care clinical settings, as many as 50–70% of AD cases are misdiagnosed^23^. Diagnoses conducted by expert, specialized clinicians are considerably better, but may still misdiagnose 20–30% of patients^23–25^. Furthermore, several clinical trials found that 16–23% of patients enrolled with clinical diagnoses of AD lacked evidence of amyloid pathology^24^. Diagnosing AD by the presence of amyloid plaques can also be problematic, as a third of elderly CU individuals may also have positive amyloid scans^26^. Further confounding AD diagnoses, decreased CSF levels of Aβ are not observed solely with AD; patients with vascular dementia, Creutzfeldt–Jakob disease (CJD), Lewy Body Dementia (LBD), and Frontal Temporal Lobar Dementia (FTLD) can have low Aβ levels^27,28^. Healthy individuals may also have relatively low total Aβ peptide concentrations and can be misdiagnosed as having pathologically low Aβ42^28^. Other assays to detect soluble Aβ oligomers, not based on α-sheet conformation, report discrimination of AD and age-matched controls, however, there is considerable overlap between the levels of Aβ oligomers between the groups that precludes distinct differentiation based on the biomarker alone^29,30^. These challenges in AD diagnosis suggest there is still a need for a biomarker that improves diagnostic discrimination for clinicians treating patients presenting with cognitive impairment and dementia.

We previously demonstrated that de novo designed α-sheet peptides can serve as a capture agent for the detection of toxic soluble Aβ oligomers in both CSF and plasma of AD patients^21,22^. Our assay, Soluble Oligomer Binding Assay-Alzheimer’s Disease (SOBA-AD), previously provided excellent discrimination in plasma between cognitively unimpaired (CU) individuals and patients on the AD continuum with sensitivity and specificity of 99% relative to clinical and neuropathological diagnoses^22^. SOBA-AD detects α-sheet-containing Aβ toxic oligomers, thereby allowing for discrimination of AD patients from those with other neurodegenerative diseases, including Huntington’s disease, FTLD, Progressive Supranuclear Palsy (PSP), Parkinson’s Disease (PD) and LBD^22^. The discrimination is inherent to the design of the assay.

SOBA is an ELISA-like sandwich assay, but instead of a capture antibody, we use an α-sheet peptide as the capture agent. The α-sheet capture agent binds selectively to α-sheet in the toxic oligomers. A conventional anti-Aβ antibody is used to confirm that the bound oligomers contain Aβ. Other toxic α-sheet oligomers may be present in the sample, but they are not detected unless an antibody specific for the protein being captured is employed. Furthermore, SOBA-AD was able to identify CU controls that converted to mild cognitive impairment (MCI) associated with AD prior to presentation of clinical symptoms^22^. In this case, follow-up autopsy or clinical results were available for 10 of the 11 SOBA-AD positive CU subjects (representing 12 of 13 samples), and all 10 individuals later proceeded to Mild Cognitive Impairment (MCI), suggesting that SOBA-AD is capable of early detection of AD^22^.

Our previous work described the development and testing of SOBA-AD in 379 banked cross-sectional and longitudinal AD plasma samples from the Alzheimer’s Disease Research Center (ADRC) and Behavioral Neurosciences Group (BNG) Sample and Data Repository^22^. Here, we increased testing of SOBA-AD with another 265 samples from different cohorts and with consideration for diversity. Banked samples were provided by the National Centralized Repository for Alzheimer’s and Other Dementias (NCRAD) (n = 205) and purchased from a commercial source, PrecisionMed, LLC (n = 60). Here we describe testing of these plasma samples from subjects with AD diagnoses and CU controls by SOBA-AD at the University of Washington (UW) and at AltPep Corporation (“AltPep”). We report the performance of SOBA-AD with respect to discrimination of AD patients from CU controls by detecting α-sheet containing Aβ oligomers in plasma from two independent cohorts performed at two different sites.

## Results

### SOBA-AD performance on NCRAD cohort

**Figure 1:**
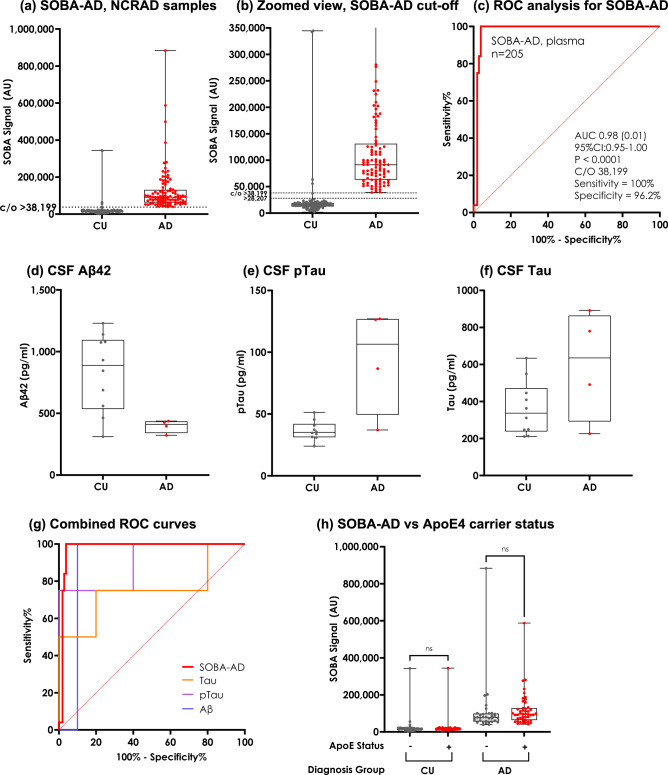
SOBA-AD signals and limited CSF biomarkers for AD and CU samples from NCRAD. (**a**) SOBA assay discriminates AD patients from cognitively unimpaired subjects (plasma samples, n = 205) with values binned by diagnosis. Diagnoses were provided by NCRAD. (**b**) The data from panel A are presented but omitting the values above 350,000 for a better view of the luminescence signal cutoff > 38,199 for determining SOBA positive cases. Note that there are 4 CU samples with SOBA values above the cutoff. ROC analysis prefers a higher cutoff; however, the distribution of points also fits a lower cutoff of > 28,207 with an additional CU sample as SOBA-AD positive. For the NCRAD cohort, the SOBA assay was performed at the University of Washington. (**c**) ROC analysis and associated results for SOBA-screened plasma in NCRAD cohort, n = 205 samples. (**d**–**f**) CSF biomarkers, Aβ42, pTau, and Tau, were available for 14 subjects (10 CU and 4 AD). Biomarker levels were plotted and binned by clinical diagnosis. (**g**) Receiver operator curves for CSF Aβ42 (AUC = 0.90, p = 0.024), CSF pTau (AUC = 0.90, p = 0.024), and CSF Tau (AUC = 0.75, p = 0.15) plotted along with SOBA-AD (AUC = 0.98, p < 0.0001) in the NCRAD cohort. AUC and significance are also reported in Table 2. Note that CSF biomarkers were only available for 14 subjects. (**h**) SOBA values within each clinical diagnosis group were independent of *APOE* ε4 carrier status (*p* = 0.2365 and *p* = 0.0526 for CU and AD groups, respectively). 167 (83%) had *APOE* genotype information available. 33% of the CU group and 47% of the AD group were carriers of at least one copy of the *APOE* ε4 allele.

We tested 205 blinded plasma samples provided by NCRAD at UW (200 subject samples and 5 pooled control samples). SOBA-AD provided good discrimination between CU and AD subjects (sensitivity, specificity, and area under the curve of 100%, 96%, and 98%, respectively) with a cutoff luminescence signal of 38,199, as determined through a ROC analysis (95% CI 0.95–1.00) (Fig. 1a–c). The effect size, as measured by Cohen’s *d*, was *d* = 1.16, indicating a large effect. Four CU samples had SOBA values above the luminescence cutoff (“SOBA-AD positive”) and did not agree with the clinical classification provided by NCRAD. Though ROC analysis favored a higher cutoff, the distribution of points also suited a lower cutoff of 28,207, consistent with the cutoff used in our previous study using the same plate reader but under a different antibody system (6E10 with a secondary antibody versus 6E10 with covalent attachment of the horseradish peroxidase, HRP, necessary for signal detection)^22^. Analyzing SOBA-AD performance using the lower cutoff added an additional SOBA-AD positive CU sample and lowered specificity from 96 to 95% (Fig. 1b). This lower cutoff was used in reporting the blinded results to NCRAD.

**Figure 2:**
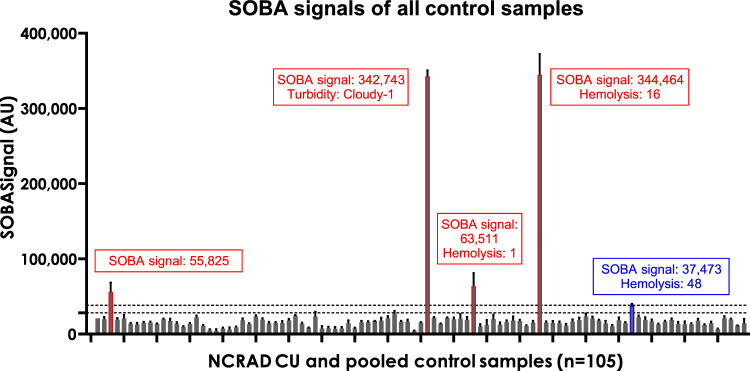
SOBA signals for CU control samples highlighting sample integrity issues of 4 samples above the SOBA cutoff value of 38,199. 4 CU samples had SOBA values above the cutoff of 38,199, suggesting positivity for toxic oligomers (samples highlighted in red). Sample integrity issues, including hemolysis and turbidity, were observed in 3 of the 4 false-positive samples. However, sample integrity is unlikely to be a pivotal factor in the high SOBA values for the false positives given that turbidity was observed in 2 other CU samples (3 total) (of a range 1–2) and hemolysis was observed in 48 other CU and 3 pooled controls. Using a lower cutoff of 28,207, an additional SOBA-AD positive CU sample was considered (highlighted in blue) with hemolysis grade of 48.

We examined whether sample integrity contributed to false-positive results (Fig. 2). Upon examination, 53% of the entire NCRAD cohort had a non-zero degree of turbidity or hemolysis, including 53 CU samples, 53 AD samples, and 3 pooled controls (Supplemental Table S1). Though 4 of the 5 potential false-positive samples presented hemolysis or a turbidity grade of cloudy, it was unlikely that sample integrity alone contributed to false positives for the 5 CU samples given the high number of SOBA results that correctly identified the clinical diagnoses. We previously found samples from cognitively normal subjects with α-sheet toxic oligomers as detected by SOBA-AD that later converted to mild cognitive impairment (MCI) or AD, confirmed by clinical evaluation or neuropathology^22^. Unfortunately, clinical follow up for the 5 CU individuals who tested positive by SOBA-AD is not available.

Conventional biomarkers and imaging were available for a limited number of subjects in this cohort. Amyloid PET status was available for 7 subjects in the NCRAD cohort: 5 were PET positive and two were PET negative. SOBA-AD correlated with PET status for 6 of 7 subjects (Table 1). All 5 of the AD cases with elevated PET were also SOBA-AD positive. For the two CU cases, one was both PET and SOBA-AD negative. In the discordant case, the subject was PET positive, but SOBA-AD negative and clinically diagnosed as CU with a CDR-SUM score of 0.

**Table 1:** Correlation of PET status, SOBA-AD, and clinical diagnosis in NCRAD cohort.

Clinical diagnosis	SOBA-AD signal (AU)	SOBA-AD status	PET status
AD	280,611	Positive	Elevated
AD	80,618	Positive	Elevated
AD	64,325	Positive	Elevated
AD	63,059	Positive	Elevated
AD	51,365	Positive	Elevated
CU	20,843	Negative	Elevated
CU	15,253	Negative	Normal

Only 7 subjects in the NCRAD cohort had PET imaging. Both SOBA-AD cutoffs (38,199 and 28,207) are based on receiver operator curve analyses.

CSF Aβ42, phosphorylated Tau (pTau), and total Tau levels were available for only 14 of the 200 subjects, and they are binned by diagnosis (Fig. 1d–f). Despite the limited number of samples, we performed a ROC analysis to compare SOBA-AD performance with the conventional biomarkers. SOBA-AD showed improved discrimination of AD and CU subjects (Fig. 1g, Table 2); although, CSF Aβ42 and CSF pTau had AUC of 0.90, these results were available for only 4 of the 100 AD patients. The SOBA-AD levels were correlated with CSF pTau biomarker levels (r = 0.66, p = 0.0089) but not with CSF Aβ42 and total Tau (Table 3).

**Table 2:** Receiver operator curve analyses of SOBA-AD compared to CSF biomarkers in NCRAD and PrecisionMed cohorts.

NCRAD cohort	SOBA-AD	Aβ42^a^				pTau^a^	Total Tau^a^
AUC (SD)	0.98 (0.01)	0.90 (0.095)				0.90 (0.10)	0.75 (0.18)
95% CI	0.95–1.00	0.71–1.00				0.70–1.00	0.41–1.00
*p*-value	< 0.0001	0.0237				0.0237	0.1573

PrecisionMed cohort	SOBA-AD	Aβ42	Aβ40	Aβ42/40	Aβ38		Total Tau^b^
AUC (SD)	1.00 (0.00)	0.54 (0.075)	0.56 (0.075)	0.64 (0.071)	0.63 (0.072)		0.86 (0.047)
95% CI	1.00–1.00	0.39–0.68	0.41–0.70	0.50–0.78	0.49–0.77		0.77–0.96
*p*-value	< 0.0001	0.6152	0.4420	0.0625	0.0837		< 0.0001

^a^Only 14 of 200 plasma samples in the NCRAD cohort had associated CSF biomarker levels.

^b^57 of 60 plasma samples in the PrecisionMed cohort had associated CSF Tau levels reported.

**Table 3:** Correlation of SOBA-AD to CSF biomarkers in NCRAD and PrecisionMed cohorts.

NCRAD cohort	Aβ42^a^			pTau^a^	Total Tau^a^
Pearson’s r	− 0.45			0.66	0.519
*p*-value	0.10			0.0089	0.057

PrecisionMed cohort	Aβ42	Aβ40	Aβ42/40	Aβ38	Total Tau^b^
Pearson’s r	− 0.13	0.054	− 0.24	0.11	0.35
*p*-value	0.34	0.68	0.063	0.40	0.0081

^a^Only 14 of 200 plasma samples in the NCRAD cohort had associated CSF biomarker levels.

^b^57 of 60 plasma samples in the PrecisionMed cohort had associated CSF total Tau levels reported.

As Apolipoprotein E4 (*APOE* ε4) status is a risk factor for AD, we evaluated α-sheet toxic oligomers levels, as measured by SOBA-AD, with respect to carrier status. SOBA levels were not significantly correlated with *APOE* ε4 carrier status for both CU (p = 0.2365) and AD (p = 0.0526) (Fig. 1h). However, given the trend for AD, four outliers (one from each group with SOBA values above 300,000) were removed from the analysis; carrier status remained independent for the CU group (p = 0.2595) but was significant for the AD group (p = 0.0363). SOBA-AD also had a negative correlation with age of subjects for the CU group (*r* = − 0.30, *p* = 0.0022) (SI Fig. 1a). When the five potential false positives (CU by clinical diagnosis, SOBA-AD positive) were excluded from the analysis, the negative correlation for the CU group remained unchanged (*r* = − 0.32, *p* = 0.0015). SOBA-AD was not significantly correlated with sex, race, or CDR-SUM scores for either CU or AD (SI Fig. 1b–d).

### SOBA-AD performance on PrecisionMed cohort

**Figure 3:**
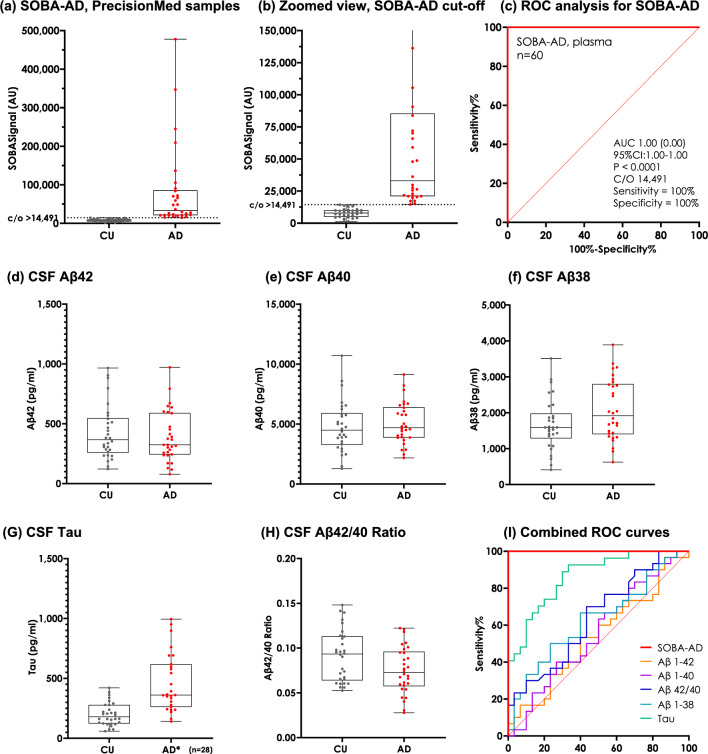
SOBA signals and CSF biomarkers for AD and CU samples from PrecisionMed. (**a**) Samples purchased commercially from PrecisionMed were screened by the SOBA assay at AltPep. Values here are binned by AD and CU as designated by Precision Medicine. The cutoff for SOBA positivity was luminescence signals > 14,491. (**b**) The same data as in panel A are presented but values over 150,000 are not shown to better view the cutoff region. (**c**) Receiver operator curve analysis and associated results for the SOBA-screened plasma samples from PrecisionMed relative to the clinical diagnoses. (**d**–**h**) Aβ42, Aβ40, Aβ38, Tau, and Aβ42/40 ratio CSF biomarkers exhibit significant overlap making it difficult to distinguish AD from CU. Values here are binned by AD and CU as designated by PrecisionMed. (**i**) Receiver operator curves were constructed relative to the clinical diagnoses for SOBA-AD (red) and compared to the CSF biomarkers: Aβ42 (AUC = 0.54, p = 0.62), CSF Aβ40 (AUC = 0.56, p = 0.44), CSF Aβ42/40 (AUC = 0.64, p = 0.063), CSF Aβ38 (AUC = 0.63, p = 0.084), and CSF Tau (AUC = 0.86, p < 0.0001). The AUC values and statistical significance are also reported in Table 2. Note that 3 AD subjects were missing Tau levels.

To further investigate SOBA-AD on another cohort, PrecisionMed samples were evaluated at AltPep utilizing similar but not identical reagents and instrumentation. The assay was performed blindly by a certified Medical Laboratory Scientist. SOBA-AD provided discrimination of the CU and AD diagnosis groups with no overlap (Fig. 3a,b). ROC analysis relative to the clinical diagnoses resulted in a SOBA luminescence cutoff of 14,491 and 100% sensitivity and specificity (95% CI 1.00–1.00) (Fig. 3c). The effect size, as measured by Cohen’s *d*, was *d* = 0.96, indicating a large effect. The absolute SOBA values, which are relative luminescence signals, were lower for the PrecisionMed cohort than the NCRAD cohort due to instrumentation differences. Specifically, the plate readers at UW and AltPep are from different manufacturers and had different gain values—the UW Perkin Elmer plate reader software automatically calculates the gain, expanding the scale, whereas the AltPep Biotek plate reader software allows for user-defined gain for improved day-to-day comparisons. Though the relative signal intensities for SOBA-AD differed, the trend within the samples remained consistent with high SOBA signals correlating with AD classification.

Though all 30 AD subjects underwent MRI or CT scans to rule out other causes of their clinical symptoms, amyloid PET imaging was only available for two PrecisionMed subjects. Both subjects were PET positive, consistent with their clinical diagnosis of AD, and they were also SOBA-AD positive. Standard CSF biomarkers, Aβ42, Aβ40, Aβ38, and Tau were provided by PrecisionMed for all participants in the cohort with the exception of CSF Tau, which was reported for 95% (57 of 60) of the subjects. These biomarker levels were plotted and binned by diagnosis group, resulting in significant overlaps between CU and AD groups (Fig. 3d–h). Concordance of the individual CSF biomarkers with clinical diagnosis are presented in Table 2. SOBA-AD provided higher sensitivity, specificity, and AUC by ROC analysis compared to the associated CSF biomarkers for the plasma samples in the PrecisionMed cohort (Fig. 3i and Table 2). In contrast to CSF biomarkers, plasma SOBA-AD discriminated AD and CU subjects with no overlap (Fig. 3b). There were no significant correlations when comparing age, sex, race, and cognitive scores with the SOBA-AD signal across diagnosis groups in the PrecisionMed cohort (SI Fig. 2). SOBA-AD was correlated with CSF Tau (r = 0.35, p = 0.0081) but not the other CSF biomarkers (Table 3).

### Toxic oligomers are abundant in AD patients and not in CU controls

**Figure 4:**
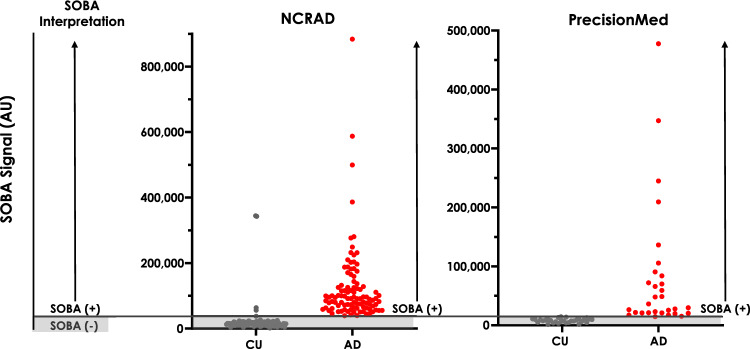
Interpretation of SOBA-AD positive samples above a defined cutoff. SOBA-AD positive samples were defined as having raw luminescence values above the cutoff. Relative signal intensities for SOBA-AD differed between the two cohorts due to differences in plate readers and gain settings, as described in the text.

SOBA-AD was performed on 265 samples sourced from two independent cohorts using two different laboratory settings, including instrumentation, personnel, and location. Consistent with previously reported SOBA-AD performance, SOBA-AD provided discrimination of AD and CU subjects in both the NCRAD and PrecisionMed cohorts (Table 4). High toxic oligomer burden was found in AD patients but not in CU controls. Diagnosis of AD was strongly correlated with a SOBA-AD positive readout (Fig. 4).

**Table 4:** Overall performance of SOBA-AD on NCRAD and PrecisionMed cohorts.

Cohort (n, CU/AD)	NCRAD (205, 105/100)	PrecisionMed (60, 30/30)
Agreement with clinical diagnosis (%)	98%	100%
Sensitivity	100%	100%
Specificity	96%	100%

## Discussion

Here we evaluated SOBA-AD performance in 265 plasma samples, from 260 subjects plus 5 control samples of plasma pooled from CU subjects, and from two independent cohorts. SOBA-AD discriminated clinically diagnosed AD patients from CU controls with high sensitivity and specificity. In contrast to targets in healthy individuals, α-sheet-containing Aβ oligomers are abundant only in those with the disease, leading to separation of SOBA signals for CU and AD samples. Consequently, SOBA-AD differentiated AD from controls with minimal to no overlap, demonstrating consistent performance in a combined set of 644 samples collected and processed from 3 cohorts (379 reported previously^22^).

The evaluation of the two cohorts presented here took place at two different laboratory sites with similar but not identical reagents and instrumentation. The antibody system used to detect captured α-sheet containing Aβ oligomers differed from the previously reported system^22^ in that the primary 6E10 antibody was conjugated with horseradish peroxidase (HRP) to enable direct quantification without the need of a separate secondary antibody. SOBA-AD performance was consistent across the 3 cohorts despite these variables. Combined with our previously reported work^22^, SOBA-AD discriminates AD from CU controls with ranges of sensitivity from 99 to 100%, specificity from 95 to 100%, and AUC from 98 to 100%.

We examined sample integrity as a potential source of disagreement between SOBA-AD positivity and an CU clinical diagnosis in the NCRAD group but determined that was unlikely to be the cause of the discrepancy. For the 4–5 (depending on signal cutoff used) discordant samples, the soluble oligomers appear to be robust enough to be unperturbed by common sample processing variations and factors such as hemolysis and lipemia. It is possible the false-positive samples reflect subjects who are incubating toxic soluble oligomers prior to presentation of clinical symptoms. In our previous work, clinical and neuropathological follow was available for 12 of 13 SOBA-AD positive CU samples, and the individuals later progressed to MCI/AD^22^. These results suggest the potential for preclinical detection of AD by SOBA-AD, but unfortunately, we do not have follow-up information for the CU false-positive cases in the current study.

Here SOBA-AD was evaluated against clinical diagnoses as well as the available PET data and CSF biomarkers (discussed further below). Admittedly, current clinical diagnosis of AD in practice is complex and multifaceted. Recent successes in amyloid-reducing interventions such as Lecanemab (BioArctic/Eisai/Biogen)^31^ and Donanemab (Eli Lilly)^32,33^ have brought to light the need for differential biomarkers to selectively enroll and stratify AD patients who will benefit from the treatments. Many view PET imaging as the premortem standard for determining amyloid pathology; however, recent clinical trials, including those of Lecanemab and Donanemab, have used CSF biomarkers in conjunction with imaging to confirm amyloid positivity^31–33^. Furthermore, FDA approvals for Fujirebio Lumipulse^®^ G β-Amyloid (1–42/1–40) CSF and Roche Elecsys^®^ β-Amyloid(1–42), pTau(181P), and total Tau (tTau) as In Vitro Diagnostic (IVD) tests have opened the possibility for more widespread use of CSF-based biomarker testing^34,35^. Their clinical validation was established based on assay agreement with PET amyloid plaque status.

We also explored how well the conventional amyloid biomarkers agreed with the clinical diagnoses. Unfortunately, in the case of the NCRAD cohort, amyloid biomarkers are sparse: CSF Aβ42 levels were available for 14 subjects. If we define the cutoff as 450 pg/ml by ROC analysis, then there is a good separation between CU and AD with only one CU case below the cutoff. Comparing SOBA-AD and the diagnosis based on this Aβ42 cutoff, there is very good agreement, aside from a single CU subject with low Aβ42 concentration and no evidence of toxic oligomers; however, given that these data represent only 6.8% of the cohort, the analysis is not robust.

In contrast, Aβ42, Aβ40, and Aβ42/40 results were available for all subjects in the PrecisionMed cohort. These CSF biomarkers exhibit significant overlap between the CU and AD groups (Fig. 3d–h) such that they were likely not strictly used in the diagnosis, as if they had, the discordance would have been very high with Aβ42/40 at odds with the observed symptoms in 37 or 43% of the group, depending on the cutoff value used (ROC analysis or Aβ42/40 ratios utilized by FDA-approved diagnostic assays^34^, respectively). Considering CSF Aβ42/40 as an indicator for amyloid pathology, the sensitivity/specificity with respect to clinical diagnosis in the PrecisionMed cohort were 70%/57%, respectively, by ROC analysis (p = 0.0625). By the same analysis, plasma SOBA-AD yielded sensitivity/specificity of 100%/100% (p < 0.0001) (Fig. 3i). In future studies we will compare SOBA-AD values with PET imaging, as another validation metric.

Though there were a limited number of subjects with PET results in the cohorts evaluated here, SOBA-AD agreed with PET status in 8 of the 9 available cases. Extensive evidence points to low molecular weight Aβ oligomers as the species that confer neurotoxicity^1–3,22,36–42^; as a protective mechanism, the oligomers change structure^22^ and are sequestered and deposited in plaques^43^. In related diseases linked to protein misfolding, promoting the aggregation of misfolded Huntintin (Htt) and alpha-synuclein (α-synuclein) to form inclusion bodies rescues proteasome dysfunction and reverses toxicity in cell models of Huntington’s disease and Parkinson’s disease^44^. In the single discordant case here where the subject was SOBA-AD negative with a clinical diagnosis of CU but elevated PET status, it is possible that the lack of toxic oligomers and lack of cognitive symptoms is due to early plaque deposition serving as a sink for toxic Aβ. Since the PET imaging reflects amyloid plaques, it is possible for a subject with elevated PET to have amyloid plaques but low toxic oligomer levels and retention of normal cognitive function. In fact, the applicability of amyloid PET imaging in the context of AD diagnosis becomes more complicated with age, whereby 20–30% of CU older adults have Aβ plaque deposition^45^. Without longitudinal follow up, we cannot determine whether such discordant cases are examples of preclinical, non-symptomatic AD.

Moving forward, the AD scientific and clinical communities look to define AD biologically at the molecular level and determine how and when that relates to the clinical definition. Biomarkers from imaging, CSF, and plasma analyses can be useful in delineating clinical stage, progression, and treatment response. Owing in part to major observational and research cohorts like ADNI (Alzheimer’s Disease Neuroimaging Initiative), Swedish BioFINDER (Biomarkers For Identifying Neurodegenerative Disorders Early and Reliably), AIBL (The Australian Imaging, Biomarker & Lifestyle Flagship Study of Ageing), and many other notable studies, the performance and relevance of biomarkers have been extensively validated and utilized in clinical trials and clinical practice^46–50^. It is now possible to identify disease at an asymptomatic preclinical stage^23,51–57^. Biomarkers in CSF and plasma such as pTau217, pTau181, pTau231, Glial Fibrillary Acidic Protein (GFAP), and Neurofilament Light Chain (NfL) have shown consistency across different cohorts with strong association with amyloid and tau pathology by PET imaging and postmortem pathology, as well as being associated with reduced clinical progression in disease-modifying clinical trials^31,32,58–76^. Following these developments, the National Institute of Aging and the Alzheimer’s Association (NIA-AA) moved to revise the diagnostic guidelines to define AD biologically with the inclusion of biomarkers, reflecting confidence in the performance of plasma and CSF biomarkers as measures of the core pathophysiology of AD^77^. The use of CSF and plasma biomarkers has great potential for rapid screening, reducing the dependence on PET scans, and facilitating recruitment of participants in clinical trials and identification of patients for treatment at an early stage of disease, particularly for those patients presenting sub-threshold levels of amyloid pathology by PET. In principle, as a simple blood-based test, SOBA-AD fits this paradigm by providing separation of AD and non-AD populations, and it has been shown it can detect earlier stages of the disease.

Despite several research reports describing and targeting soluble Aβ oligomers for AD detection and disease-modifying treatments, α-sheet containing oligomers remain a novel target. Other groups have tried to detect and examine the relevance of Aβ oligomers primarily based on antibodies targeting molecular size and solubility. However, these approaches fail to cleanly discriminate patient samples from healthy controls, due to lack of distinction in response between the relevant oligomers and other aggregated forms of Aβ^29,30,78^. Furthermore, treatment-associated cognitive benefits from drugs targeting oligomeric forms of Aβ by binding sequence-specific epitopes, is still unproven^79,80^. We believe this is because the α-sheet structure is a highly specific and a defining feature of toxic oligomers and our de novo α-sheet detection agent, unlike antibodies, discriminates based on secondary structure, not side chain epitopes, which are present in multiple conformations and aggregate forms of Aβ.

The banked samples used in our previous study contained primarily Caucasian samples^22^. In this report, we aimed to increase testing of SOBA-AD on additional cohorts with consideration for diversity. We obtained as many samples from ethnic and racial minorities as were available from PrecisionMed. Similarly, we requested any available non-Caucasian samples from NCRAD. As a result of these efforts, there were 21% and 37% of non-Caucasians in the NCRAD and PrecisionMed cohorts, respectively. Based on this still limited sampling, SOBA-AD agreed with the clinical diagnoses independent of race. Compared to non-Hispanic Caucasians, Black and Hispanic individuals have a higher risk of AD and bear a disproportionate burden of disease^81–84^. Even so, they have been underrepresented in AD clinical trials and likely in estimates of the prevalence of AD^85–87^. Moreover, both Lecanemab and Donanemab clinical trial recruitment efforts saw Black and Hispanic symptomatic volunteers screened out by amyloid threshold requirements at higher rates than non-Hispanic Caucasians^87^. Continued intentional recruitment of samples from diverse populations is improving and will be essential to further our understanding of α-sheet toxic oligomers and their role in the pathophysiology and diagnosis of AD.

## Conclusions

We have extended plasma testing with SOBA-AD with 265 samples from two independent cohorts performed at two different sites. SOBA-AD provided discrete separation and discrimination of AD patient plasma samples from cognitively unimpaired control plasma samples with 100% sensitivity, 95–100% specificity, and 98–100% AUC across two cohorts. We note that the 4–5 (depending on signal cutoff) false positives may represent CU subjects in the earliest stages of AD, as we observed and were able to validate in a previous study^22^. The findings presented here are consistent with those published previously^22^ despite differences in reagents, instrumentation, sites, personnel, sample sources, and populations, supporting SOBA-AD as a promising test to aid in diagnosing AD.

## Methods

### Cohort 1: NCRAD cohort

205 EDTA-treated plasma samples from 200 subjects and 5 pooled controls were obtained from the National Centralized Repository for Alzheimer’s and Other Dementias (NCRAD), referred to as the NCRAD cohort. NCRAD, funded by the National Institutes on Aging (NIA), maintains clinical information and biological materials from individuals with Alzheimer’s disease and/or related dementias, as well as healthy control subjects. Subject consent, ethics review, and approval were obtained and managed by nine Alzheimer’s Disease Research Centers (ADRCs) with institutional review boards and ethics committees for each site (NYU, OHSU, Boston University, UT Southwestern, Washington University, Arizona University, Massachusetts General Hospital, University of Michigan, and University of Pittsburgh.) Deidentified samples were banked at NCRAD biorepository as approved by Indiana University IRB. Request and access to the samples were managed and coordinated by NCRAD Biospecimen Review Committee, from whom the NCRAD samples in this study were requested. The protocols are described in detail on their website: https://ncrad.iu.edu. All research was performed in accordance with relevant guidelines and regulations. Subjects were selected for an equal number of cognitively unimpaired (CU) and AD subjects, with associated PET imaging results described if available. At the time of sampling, CU subjects had no history or family history of neuropsychiatric or neurodegenerative disease and presented with no neurological complaints. Additionally, 5 of the plasma samples were pooled healthy controls, provided as part of the blinded samples by NCRAD. We requested as many non-Caucasian samples as possible. For each of the 200 subjects, two 200 µL tubes of EDTA-treated plasma were received by UW from NCRAD and stored at − 80 °C. The two sample tubes were thawed at 37 °C for 2 min and immediately placed on ice, then combined into Eppendorf Protein LoBind tubes (Cat No. 13-698-794 from Fisher Scientific) and centrifuged at 4300*g* for 10 min at 4 °C to remove platelets and insoluble precipitated material. The supernatant was transferred to a separate Protein LoBind tube using a glass pipette, and the pooled material was tested by SOBA-AD at UW. These samples were evaluated in triplicate except for 8 samples that were run as duplicates and two run as singlets due to limited plasma volume.

### Cohort 2: PrecisionMed cohort

60 commercial EDTA-treated plasma samples from 60 subjects were obtained from PrecisionMed (Carlsbad, California), referred to as the PrecisionMed cohort. Sample collection was approved by Western Institutional Review Board for PrecisionMed (protocols 8009 and 8200 for AD and healthy controls, respectively). Subject consent was also obtained and managed by PrecisionMed. All research was performed in accordance with relevant guidelines and regulations. To achieve broader diversity, subject race and ethnicity were a consideration for sample selection. An equal number of EDTA-treated plasma samples from healthy controls (cognitively unimpaired) and AD subjects were selected (30 CU and 30 AD samples). 1 mL aliquots were received and stored at − 80 °C before testing by SOBA-AD at AltPep. For testing, the full 1 mL sample was thawed at 37 °C for 2 min and immediately placed on ice. Four 250 µL aliquots were taken and placed into separate Protein LoBind tubes. One tube was centrifuged at 4300*g* for 10 min at 4 °C to remove platelets and insoluble precipitate material. The other 3 tubes were immediately re-frozen at − 80 °C. The supernatant was transferred to a separate Protein LoBind tube using a glass pipette and tested using SOBA-AD, in duplicate.

### Clinical diagnosis classification

Subject plasma samples were classified based on clinical diagnoses as cognitively unimpaired (CU) controls, and Alzheimer’s disease (AD) by the respective organizations. Samples were chosen for this validation study for an even split of CU and AD in each study with consideration for racial and ethnic diversity as well as prioritizing patients that had amyloid PET imaging results available for verification.

### CSF biomarkers

All CSF biomarker measurements for subjects in this study were provided by NCRAD and PrecisionMed where available. The PrecisionMed samples were assayed with the Meso Scale Discovery (MSD) Amyloid Beta Peptide panel for Aβ42, Aβ40, and Aβ38 as singlets. The Aβ42/40 ratio was calculated from Aβ42 and Aβ40 concentrations. The MSD Human Total Tau kit was used to measure total Tau for 56 of 57 subjects. The remaining subject with total Tau levels provided used the Lumipulse G Total Tau IVD assay.

### Subject characteristics

The characteristics of the two cohorts are provided in Table 5. The CU and AD groups in the NCRAD cohort had similar age and sex. Out of the 200 subjects, 167 (83.5%) had *APOE* genotype information available. 33% of the CU group and 47% of the AD group were carriers of at least one copy of the *APOE* ε4 allele (*p* = 0.009). The mean cognitive scores using the Clinical Dementia Rating, Sum of Boxes (CDR-SUM) scale were significantly different (*p* < 0.0001) in the two groups with scores of 0 ± 3 for CU and 6 ± 4.3 for AD subjects. Mini-Mental State Exam (MMSE) scores were not available for the majority (94%) of subjects.

**Table 5:** Subject characteristics by diagnosis groups of each study.

Cohort (n)	NCRAD (200)^a^			PrecisionMed (60)		
Diagnosis group (n)	CU (100)	AD (100)	*p*-value	CU (30)	AD (30)	*p*-value
Mean age, years (range)	73 ± 7.4 (53–91)	75 ± 9.1 (53–95)	0.2	58 ± 12.8 (21–76)	65 ± 10.0 (52–86)	0.01
Sex, female/male	52 / 48	59 / 41	0.4	15 / 15	10 / 20	0.3
MMSE ± SD (range)	28.9 ± 1.1^b^ (27–30)	14 ± 5.7^b^ (10–18)	–	30 ± 0.4 (28–30)	21 ± 3.3 (14–28)	< 0.0001
CDR-SUM ± SD (range)	0 ± 0.3 (0–2)	6 ± 4.3 (0–18)	< 0.0001	N/A	4 ± 2.0^c^ (1–9.5)	–
*APOE* ε4 status, carrier^d^	33	47	0.009	N/A	N/A	–
Race (n)						
Caucasian	70	89		19	19	
Hispanic	3	1		6	5	
Black or African American	23	6		5	4	
Asian	1	1		0	2	
Native Hawaiian or Pacific Islander	3	3		0	0	

^**a**^Note, the 5 pooled plasma CU control samples are not included here.

^b^(n = 10) for CU and (n = 2) for AD due to lack of MMSE scores for the majority of the NCRAD cohort.

^c^(n = 28) AD subjects with CDR-SUM scores provided in the PrecisionMed cohort. No CDR-SUM scores were provided for CU subjects.

^d^13 CU and 20 AD subjects were missing APOE genotype information in the NCRAD cohort. Furthermore, no genotyping information was provided for any subjects from the PrecisionMed cohort.

Continuous measures are represented as mean ± SD. Statistical significance and p-values were calculated with χ^2^ or Fisher exact tests for categorical variables and unpaired Welch’s t-test for continuous variables, where appropriate. Statistical significance was not calculated where the data did not meet the requirements of the statistical test and/or data were missing for subjects.

In the PrecisionMed cohort, the CU group was younger than the AD group by mean age (p = 0.01); the inclusion of young controls, including 7 CU subjects younger than 52 years, contributed to this difference in mean age. By design, there was a similar number of males and females in each diagnosis group. CDR-SUM scores were available for 28 of 30 AD subjects (4 ± 2.0) and none of the CU subjects. MMSE scores were available for all 60 subjects in the cohort. The PrecisionMed cohort had mean cognitive scores of 30 ± 0.4 and 21 ± 3.3 by MMSE for the CU and AD groups, respectively. The mean MMSE scores were significantly different (*p* < 0.0001).

### SOBA-AD assay protocol

The NCRAD cohort was evaluated at UW in the Daggett Lab. The PrecisionMed cohort was evaluated at AltPep. Samples were blinded in both studies. In the case of the NCRAD samples, the SOBA results were submitted to and de-blinded by NCRAD. We were then provided with anonymized diagnoses, subject characteristics, and other biomarker information corresponding to the SOBA-AD readout. SOBA-AD was performed as described previously^22^, with the exception that the Aβ-targeting primary antibody was directly conjugated with horseradish peroxidase for detection (0.03 µg/mL 6E10-HRP, Cat no. 803010, Biolegend, San Diego, CA). Furthermore, assay measurements were collected on different plate readers: PerkinElmer Enspire multimode plate reader (Waltham, MA) at UW and Biotek H1 Synergy plate reader (Winooski, VT) at AltPep. Due to instrument differences, different gain and read time settings for signal detection were used by each site. Most notably, an automatic gain function is used by the Perkin Elmer plate reader, whereas a gain setting of 190 was used for the Biotek H1 Synergy reader. As such, while both instruments provided relative luminescence signals, the absolute values differed.

### Statistical analyses

We performed standard power calculations using anticipated means derived from the control and AD groups in our previous work^22^. With 95% power and alpha of 0.01, standard power calculations resulted in a total sample size of 2. The low sample numbers are due to the separation between CU and patients on the AD continuum. Even so, for our study to have biological significance, we obtained as many samples as was reasonably feasible by funding and allowable by the sourced biobank organizations (60 plasma samples from PrecisionMed, LLC and 205 plasma samples from NCRAD).

Differences in characteristics of the subjects of CU and AD diagnosis groups within each cohort were compared using Welch’s unpaired t-test for continuous variables and χ^2^ or Fisher exact test (as appropriate) for categorical variables. The performance of SOBA-AD and standard CSF biomarkers (Aβ42, Aβ38, Aβ40, pTau, total Tau, and Aβ42/40 ratio) in predicting clinical diagnosis were evaluated using Receiver Operator Curve (ROC) analysis with 95% confidence intervals. Effect size was measured by Cohen’s *d* and a standard interpretation was applied (e.g., small = 0.2, medium = 0.5, and large = 0.8 or greater). Mann–Whitney test, Kruskal–Wallis test, or Spearman’s correlation method was used to calculate whether SOBA-AD levels were correlated with *APOE* ε4 carrier status, age, sex, race, or cognitive scores within diagnosis groups. Pearson’s correlation method was used to evaluate correlation of SOBA-AD with CSF biomarker levels. Statistical analyses were performed using GraphPad Prism and *p* < 0.05 was considered statistically significant.

### Ethics approval and consent to participate

All human subjects provided informed consent; subject consent was collected and managed by the respective organizations (NCRAD and PrecisionMed, LLC).

### Supplementary Information

Supplementary Information.

## Data Availability

The data generated and analyzed during this study are included in the body of the paper and the Supporting Information.
