# Supplementary material for: Performance of SOBA-AD blood test in discriminating Alzheimer’s disease patients from cognitively unimpaired controls in two independent cohorts
Source: Sci Rep. 2024 Apr 4;14:7946. doi: 10.1038/s41598-024-57107-w (PMC10995183; doi:10.1038/s41598-024-57107-w)
Supplement: Supplementary file 1 — Supplementary Information. [file 41598_2024_57107_MOESM1_ESM.pdf]

Supporting Information for

Performance of SOBA-AD blood test in discriminating Alzheimer's disease patients from cognitively unimpaired controls in two independent cohorts

Amy Chen, Dylan Shea, Valerie Daggett*

*correspondence: daggett@uw.edu, vdaggett@altpep.com

**Table S1: Sample integrity of NCRAD cohort**			
**Sample type (n)**	**CU (100)**	**AD (100)**	**Pooled Controls (5)**
Samples with Turbidity Grade > 0 (median, range)	3 (1, 1-1)	4 (1.5, 1-2)	--
Samples with Hemolysis Grade > 0 (median, range)	50 (27.5, 1-80)	48 (30.5, 1-79)	3 (60, 14-81)

Turbidity and hemolysis observations were provided by NCRAD after the samples were blindly tested by SOBA-AD. Turbidity observations were graded with a range of 0 – 2 (1 = slightly cloudy, 2 = cloudy). Hemolysis grades in samples received from NCRAD had a range from 0 – 80 (higher number reflecting greater degree of hemolysis).

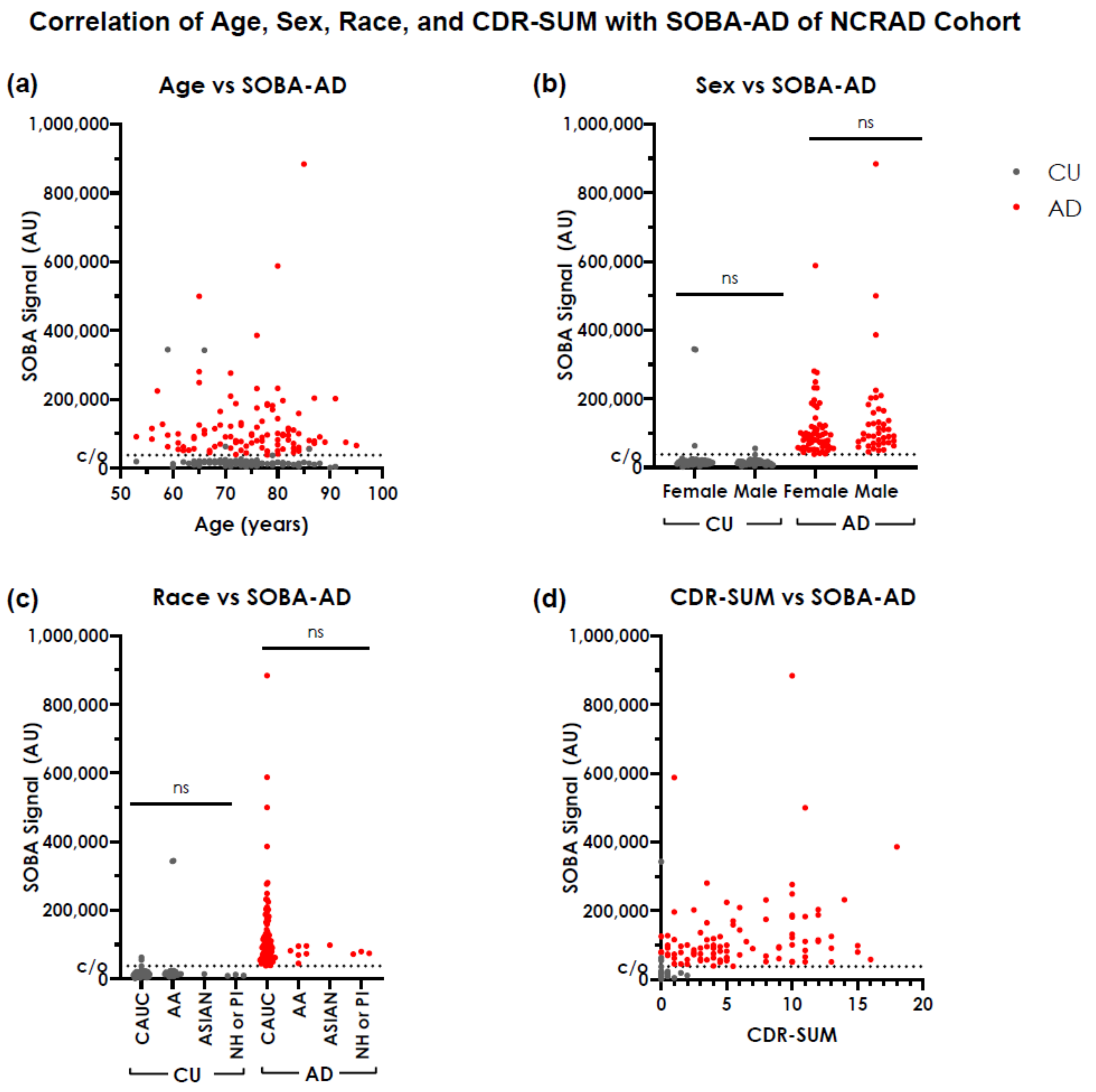

Figure S1 SOBA-AD is mostly independent of age, sex, race, and cognitive scores in the NCRAD Cohort. All 200 subject samples were plotted, binned by diagnosis group (grey dots for CU and red dots for AD) and category (for sex and race) when appropriate. A cutoff value greater than 38,199 was designated SOBA-positive. Correlations were calculated using Spearman's correlation method, Mann-Whitney test, or Kruskal-Wallis test where appropriate. (a) There was no significant correlation between SOBA-AD and age of subjects for AD (r = -0.038, p = 0.71) subjects. For CU subjects, there was a weak correlation between SOBA-AD and age (r = -0.30, p = 0.0022) and remained unchanged when five outlier points (SOBA-positive, CU samples) were

removed (r = -0.32, p = 0.0015). (b-c) No significant correlation was found between SOBA-AD and sex (p = 0.46 for CU, p = 0.12 for AD) or between SOBA-AD and race (p = 0.12 for CU, p = 0.49 for AD) for either diagnosis groups. (d) SOBA-AD did not directly reflect cognitive scores as evaluated by CDR-SUM for either diagnosis groups (r = 0.028, p = 0.78 for CU; r = 0.19, p = 0.057 for AD).

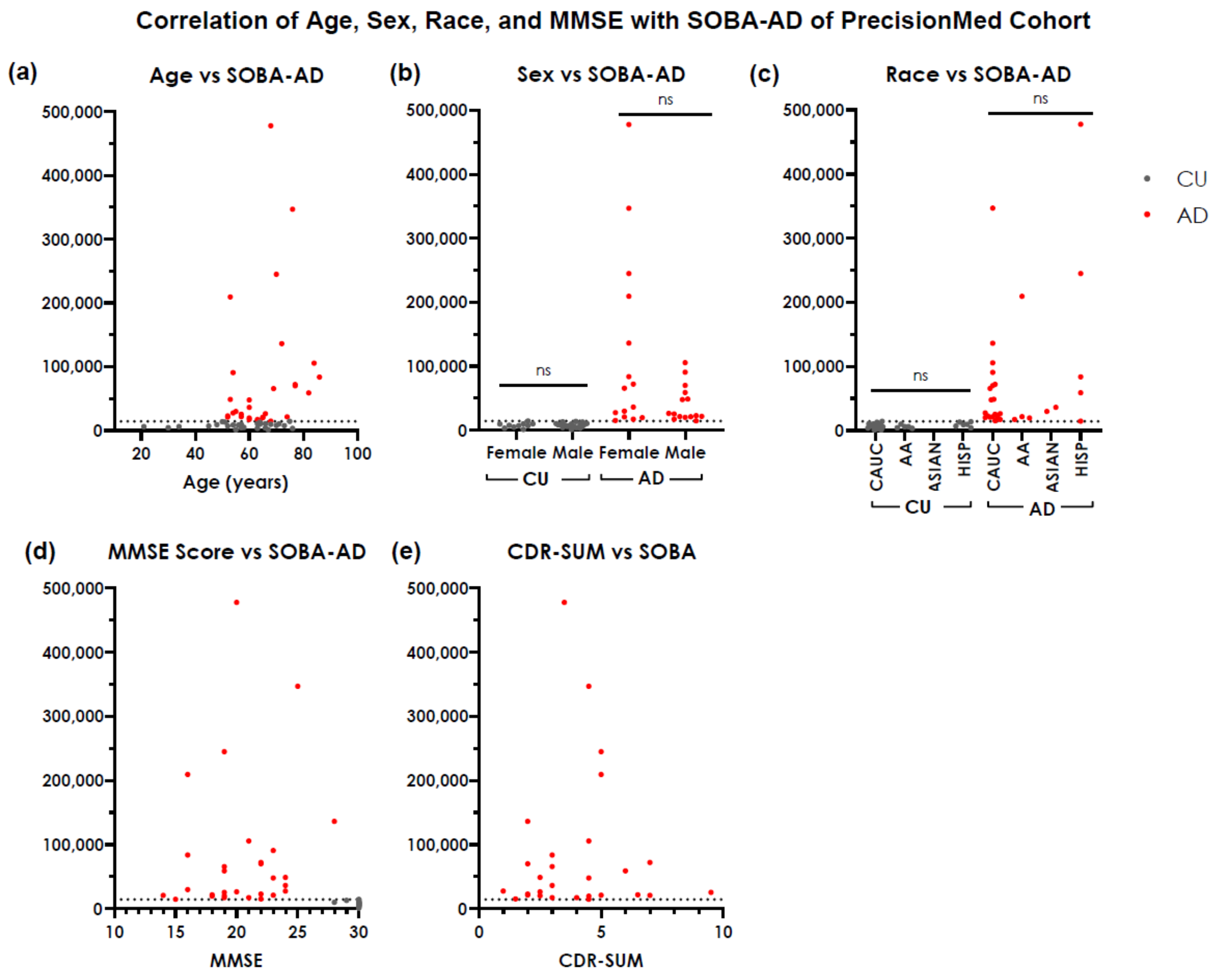

Figure S2 SOBA-AD is independent of age, sex, race, and cognitive scores in the PrecisionMed Cohort. 60 samples were plotted, binned by diagnosis group (grey dots for CU and red dots for AD) and category (for sex and race) when appropriate. A cutoff value greater than 14,491 was designated SOBA-positive. Correlations were calculated using Spearman's correlation, Mann-Whitney test, or Kruskal-Wallis test where appropriate. (a) SOBA-AD was not correlated with age of subjects for AD (r = 0.34, p = 0.071) or CU (r = 0.085, p = 0.65) subjects. (b-c) No significant correlation was found between SOBA-AD and sex (p = 0.81 for CU, p = 0.15 for AD) or between SOBA-AD and race (p = 0.26 for CU, p = 0.50 for AD) for either diagnosis groups. (d) SOBA-AD did not directly reflect cognitive scores as evaluated by MMSE (r = -0.28, p = 0.14 for CU; r = 0.25, p = 0.18 for AD) for either diagnosis groups. (e) CDR-SUM scores were only provided for 28 AD subjects; no significant correlation was found between SOBA-AD and CDR-SUM scores (r = 0.10, p = 0.60).
